# Arbitrary Inequality in Reputation Systems

**DOI:** 10.1038/srep38304

**Published:** 2016-12-20

**Authors:** Vincenz Frey, Arnout van de Rijt

**Affiliations:** 1 Department of Sociology, Utrecht University, Padualaan 14, Utrecht, 3584CH, The Netherlands; 2 Department of Sociology, Stony Brook University, 100 Nicolls Road, Stony Brook, NY 11794, USA; 3 Institute for Advanced Computational Science, Stony Brook University, 100 Nicolls Road, Stony Brook, NY 11794, USA

## Abstract

Trust is an essential condition for exchange. Large societies must substitute the trust traditionally provided through kinship and sanctions in small groups to make exchange possible. The rise of internet-supported reputation systems has been celebrated for providing trust at a global scale, enabling the massive volumes of transactions between distant strangers that are characteristic of modern human societies. Here we problematize an overlooked side-effect of reputation systems: Equally trustworthy individuals may realize highly unequal exchange volumes. We report the results of a laboratory experiment that shows emergent differentiation between ex ante equivalent individuals when information on performance in past exchanges is shared. This arbitrary inequality results from cumulative advantage in the reputation-building process: Random initial distinctions grow as parties of good repute are chosen over those lacking a reputation. We conjecture that reputation systems produce artificial concentration in a wide range of markets and leave superior but untried exchange alternatives unexploited.

Trust problems hamper mutually beneficial exchange across a broad swath of contexts. Trust is an issue whenever exchange requires that one party – the “trustor” – first expose herself to the risk of abuse by the other party – the “trustee”. Abuse may involve failed delivery, compromised quality, shirking, theft, physical violence, or disclosure of sensitive information^1^,^2^,^3^. The threat of direct punishment by the trustor^4^ and the possibility that the trustor withdraws from future exchanges^5^,^6^,^7^ can mitigate the trust problem by providing incentives for trustworthiness. Legal institutions may also deter untrustworthy behavior and offer partial compensation for a trustor in the case of abuse^8^. However, in modern cross-border exchanges over the internet, these mechanisms often cannot warrant trust. Direct punishment is ineffective if the costs for the trustor are high^9^, the likelihood of repeat business is low^10^, and the cost of effective legal recourse is prohibitive so that trustees are not incentivized to honor trust.

Reputation systems can enable exchange when other mechanisms fall short^5^,^11^,^12^,^13^,^14^,^15^,^16^,^17^. Reputation systems collate information on past exchanges voluntarily shared by trustors^10^,^18^,^19^. This allows trustors to learn from the experiences of others and to selectively exchange with trustees of good repute^5^,^20^. At the same time, reputation systems help incentivize trustees to act honorably, as a bad reputation prevents future exchange^5^. While prominent historical examples of reputation systems exist^10^,^13^, recent technological advances have made the sharing of reputation information possible at minimal cost and unprecedented scale, enabling otherwise infeasible transactions across vast numbers of geographically dispersed parties.

Here we study an overlooked side-effect of reputation systems: Reputation building exhibits a form of cumulative advantage^21^,^22^,^23^,^24^,^25^,^26^,^27^,^28^,^29^,^30^, resulting in arbitrary inequality in transaction volume among trustees. To minimize the risk of abuse, trustors may avoid many trustees who lack a transaction history in favor of a single trustee who was found trustworthy before. Random initial distinctions thus grow as parties of good repute are chosen over those lacking a reputation. The unintended consequence is a “reputation cascade” that keeps increasing the reputational advantage of one party while preventing others from building a reputation. (See model in Supplementary Information.) This inequality is arbitrary when the excluded parties are no less trustworthy than the trusted party.

## Experiments

We tested the emergence of reputation cascades in a laboratory experiment. The experimental protocol was checked and approved by the IRB of Stony Brook University (CORIHS #2014-2787 F). The experiment was subsequently carried out in accordance with the approved protocol. 336 subjects played games in groups of four trustors and four trustees (Supplementary Information). A game consisted of one or more rounds and ended after each round with probability 1/6 (Supplementary Information). In every round, one of the trustors chose whether to place trust in one of the trustees or to withhold trust. A selected trustee chose whether to honor or abuse trust. Games were played in turn-taking style, with trustor 1’s turn in rounds 1, 5, etc., and trustor 2’s turn in rounds 2, 6, etc.

Game play yielded subjects points that converted to 1.5 US dollar cents. In any round a trustor withholding trust, any trustor not in turn, as well as any unchosen trustee received 30 points. Honored trust paid the trustor and chosen trustee 50 points each. Abused trust resulted in 0 points for the victimized trustor and *T* points for the abusing trustee. Games were played in two trust conditions. In the condition “Trust Problem”, *T* was 80 or 100 (Supplementary Information), so that the trustee earned a higher monetary payoff from abusing trust than from honoring trust. In the condition “No Trust Problem”, *T* was 0 points, rendering the trustee’s payoff of honoring trust higher than the payoff of trust abuse.

Games were played in three different reputation conditions. In the “Private” condition, the computer interface showed trustors only the results of their own turns (Supplementary Information), preventing cascading. In the “Partial” condition, the pair of even-numbered trustors (trustors 2 and 4) and the pair of odd-numbered trustors (trustors 1 and 3) could also see the results of each other’s turns, allowing each pair to coordinate on a focal trustworthy trustee. In the “Full” condition, trustors could see the results of all turns, making it possible for all to rally around a single trustworthy trustee. Accordingly, we expect inequality in exchange volume between trustees in the Trust Problem condition to increase from Private to Partial to Full. Furthermore, inequality should be smaller in the No Trust Problem conditions where trustors lack an incentive to avoid untried trustees.

This experimental approach has three advantages over use of observational data on reputation systems. First, the distributional extremities others have observed in situations of reputation-enabled trust^10^,^31^,^32^, while consistent with our argument, could also be caused, for instance, by trustee variability in quality, visibility, or price. In our design alternative sources of inequality are precluded through experimental control. Second, information sharing also enables forms of feedback other than reputation cascades such as information cascades^33^, social influence^20^,^27^,^29^, and increasing returns^34^. While these forms of feedback are not driven by avoidance of trust abuse, they could also generate inequality in reputation systems. Our design allows isolating reputation cascades as the inequality-generating mechanism through a comparison of behavior in the Trust Problem condition and the No Trust Problem condition. Third, the mutual exclusivity of reputation information available to even- and odd-numbered trustors in the Partial condition allows a direct demonstration of arbitrariness in trustee selection as it makes it possible for two cascades to form involving two distinct trustees.

## Results

**Figure 1:**
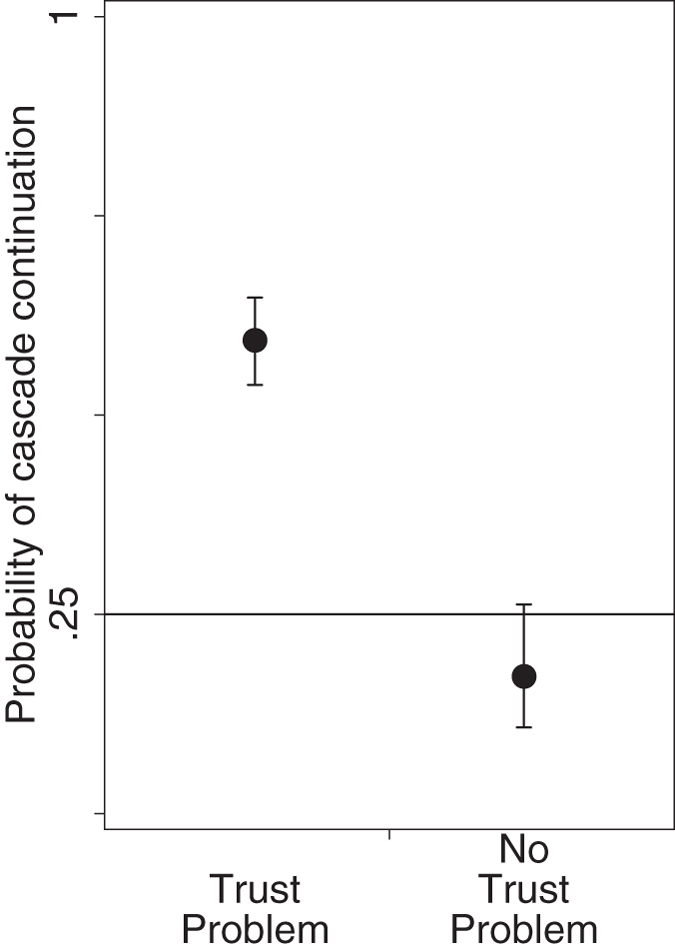
Cascading in situations with and without a threat of trust abuse. Shown is the proportion of times a trustor selected the trustee that had been selected on the preceding turn the trustor observed, given that trust had been honored. Confidence intervals are obtained from nested logistic regressions (see Supplementary Information).

We measured the prevalence of cascading as the proportion of times a trustor selected the trustee that had been selected on the most recent turn the trustor could observe, provided that trust had been honored (Fig. 1; Supplementary Information). Under the null-hypothesis that trustors randomly choose one of the four trustees every time they place trust, cascades should continue in only 25% of all cases. In the Trust Problem condition, cascades instead continued 59% of the time. This percentage increased monotonically as cascades grew in length, reaching 100% for cascades of length 5 or more (Supplementary Table 1). Remarkably, in the absence of a trust problem (Fig. 1: “No Trust Problem”) the propensity for cascading completely vanished; with 17%, cascade continuation was even lower than expected under random trustee selection. These results show that subjects formed cascades not because of shared preferences for a particular trustee identity and not because of a general tendency to imitate the choices of others, but entirely out of fear of abuse.

**Figure 2:**
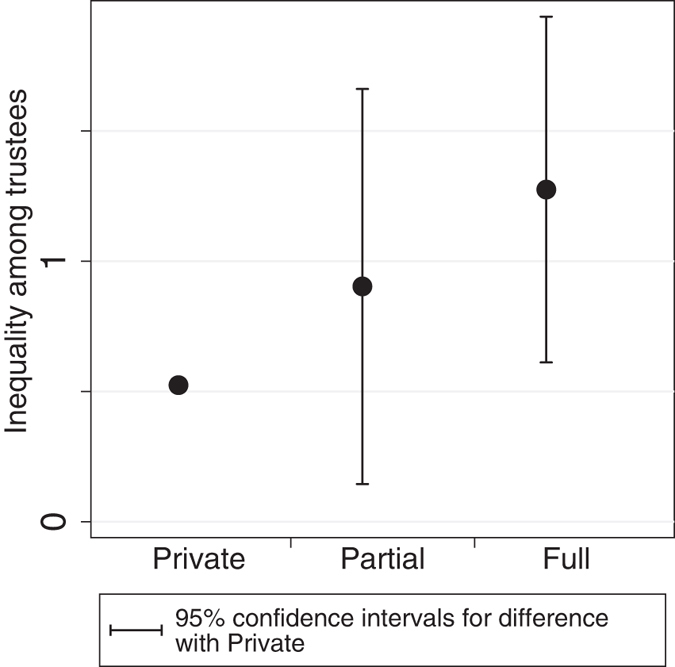
Effects of information sharing on inequality, when incentives for abuse are present (Trust Problem condition). Shown for each reputation condition is inequality among trustees in the number of times trusted in a game. 95% confidence intervals from nested linear regression models (Supplementary Information) indicate the significance of differences of Partial and Full with Private.

In the Trust Problem condition greater degrees of information sharing produced higher levels of honored trust (Supplementary Table 2), confirming earlier studies that found that information sharing facilitates exchange^9^,^10^,^35^. However, as a result of feedback in trustee selection enabled by information sharing, these gains in trust came with increased differentiation in exchange volume. Figure 2 shows that inequality in how often trustees were chosen, measured using the modified coefficient of variation^36^ (Supplementary Information), increases monotonically from Private to Partial to Full information sharing.

**Figure 3:**
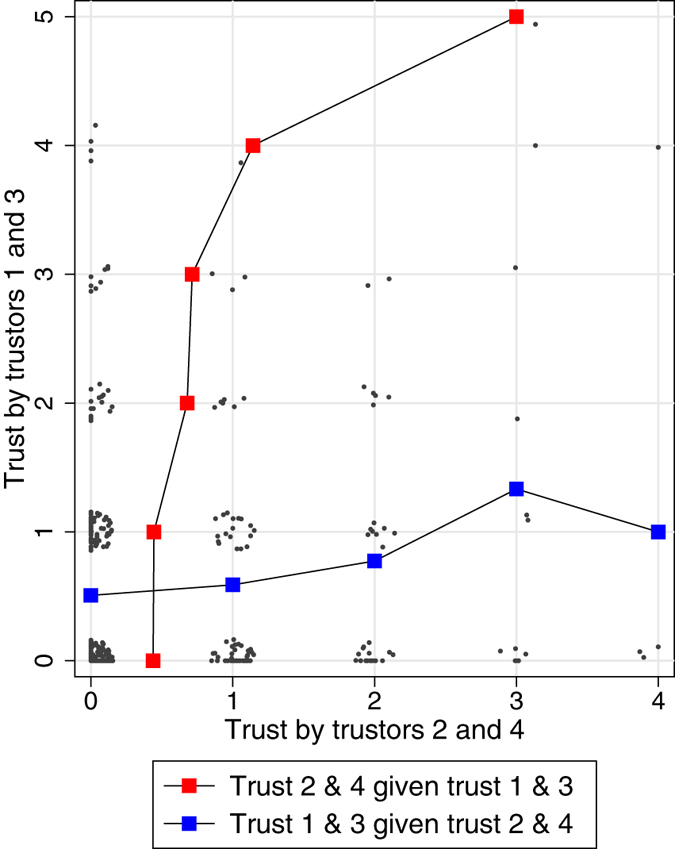
Arbitrariness of trustee selection in experimental reputation systems. Shown is the number of times a trustee was trusted by trustors 1 and 3 by the number of times that trustee was trusted by trustors 2 and 4. Data come from the Partial X Trust Problem condition, where pairs of trustors formed mutually exclusive information sharing groups and incentives for trust abuse were present.

To assess arbitrariness in trustee selection we exploited the feature of the Partial condition that the even- and odd-numbered pairs of trustors could not see one another’s choices, by comparing how often a trustee was selected by either pair. If the inequalities produced under information sharing merely reflected differences in trustworthiness across trustees, a trustee who was often chosen by the odd-numbered trustors should also have been chosen often by the even-numbered trustors, and vice-versa. Instead Fig. 3 shows that in many cases, a trustee who was often chosen by one trustor pair was never chosen by the other pair. To statistically establish arbitrariness in trustee selection we compared the disagreement in trustee choices between pairs of information-sharing trustors and pairs of non-information-sharing trustors. We find that disagreement – the difference in the number of times two trustors trusted a given trustee, summed across the four trustees – to be significantly larger among non-information-sharing trustors than among information-sharing trustors (nested linear regression, *p* = 0.007; see Supplementary Information). This demonstrates that trustees were selected or excluded from exchange based on path-dependent histories of reputation-building.

## Discussion

We conjecture that reputation cascades produce arbitrary inequality in a wide variety of everyday exchange settings that differ from the sterile conditions created in our laboratory. First, in unregulated economic exchange, established trustees can offer prices low enough to be preferable over the cheaper but riskier offers of newcomers. Laboratory experiments show that indeed trustors are willing to forgo more lucrative offers of untested parties in favor of the relative safety of exchanging in ongoing relationships with proven partners^37^,^38^,^39^,^40^,^41^. Newcomers will face even greater barriers to entry in settings where market leaders can accumulate resources that grant them greater capacity to undercut prices.

Second, our finding that trustees with longer records of trustworthy behavior were more often chosen than those with shorter records suggests that reputation cascades are robust against random deviations. While the incidental choice of an untried trustee allows a demonstration of trustworthiness, that single act does not neutralize its disadvantage in attracting subsequent trustors vis-à-vis well-established competitors. Preferences for long-standing reputations over marginal ones will likely be even stronger in everyday settings where information is not always accurate and trustees can fabricate positive ratings (compare ref. 42).

While arbitrary inequality constitutes one undesired outcome^43^,^44^,^45^, reputation cascades may have additional adverse effects. Sometimes market concentration will come with oligopolistic inefficiencies. In other instances, exchange opportunities that provide a proximity advantage or superior value are foregone^46^. Reputational feedback may also generate discrimination against groups marked by a systematic shortage of credit such as youth, innovators, and migrants. Public policy interventions – such as the EU’s prohibition of considering track records when comparing bids in public procurement or Germany’s ban on landlord requests for registries on timely payment from potential tenants – can aid in mitigating the negative consequences of reputation cascades.

## Additional Information

**How to cite this article**: Frey, V. and van de Rijt, A. Arbitrary Inequality in Reputation Systems. *Sci. Rep.* **6**, 38304; doi: 10.1038/srep38304 (2016).

**Publisher's note:** Springer Nature remains neutral with regard to jurisdictional claims in published maps and institutional affiliations.

## Supplementary Material

Supplementary Information
