# Supplementary material for: Arbitrary Inequality in Reputation Systems
Source: Sci Rep. 2016 Dec 20;6:38304. doi: 10.1038/srep38304 (PMC5171920; doi:10.1038/srep38304)
Supplement: Supplementary Information [file srep38304-s1.pdf]

Supplementary Information for: Arbitrary Inequality in Reputation Systems

Vincenz Frey*†
Arnout van de Rijt*‡§

Table of Contents

Methods

**1. The experiment**	2
1.1 Procedure	2
1.2 Computer interface	2
1.3 Instructions	4
**2. Data and analyses**	14
2.1 Visual representation	14
2.2 Estimates	14
**3. Theoretical Model**	18
3.1 The game	18
3.2 Analysis	19
3.3 Determination of experimental parameters	25
**References**	26

*ICS / Department of Sociology, Utrecht University
†To whom correspondence should be addressed. Phone: +31-30-253-1948; E-Mail: v.c.frey@uu.nl
‡Department of Sociology, Stony Brook University
§Institute for Advanced Computational Science, Stony Brook University

1 The experiment

1.1 Procedure

This experiment was conducted at the laboratory of the Center for Behavioral Political Economy at Stony Brook University in the fall of 2014. In each session of the experiment, subjects played 8 games consecutively. Each session had 24 subjects so that 3 groups of 8 subjects could be formed. For each of the 8 games, the subjects were randomly assigned to one of the 3 groups and to the role of trustor or trustee.

We organized 14 sessions. Two reputation conditions were used in each session: one in the first 4 games and another one in games 5 through 8. The trust problem condition was varied only between sessions. For each of the values $T = 80$, 100 (Trust Problem), we conducted 6 sessions, balancing the selection and order of the reputation conditions across sessions. For $T = 0$ (No Trust Problem), we conducted 2 sessions: one with the Partial condition in games 1 through 4 and the Full condition in games 5 through 8, and one with the reversed order. The No Trust Problem and Private conditions were not crossed, as this would have provided no relevant data for the theory at hand.

How many rounds the games lasted was determined in advance using a pseudo random algorithm (with a continuation probability 5/6) in order to make the length of games uniform across conditions. Subjects were informed that the length of the games was predetermined in this manner but not about the actual lengths of the games. The 8 games played in a session lasted 3, 5, 9, 5, 2, 8, 7, and 8 rounds.

In total, 336 subjects participated in the experiment (predominantly undergraduate students; average age = 20, 54% females). The sessions lasted up to about 90 minutes and subjects earned on average 23.6 US dollars.

1.2 Computer interface

Supplementary Figure 1 shows a screenshot from the Partial condition. The computer interface was inspired by that of ref. 45 and programmed in z-tree[46]. Supplementary Fig. 1 shows a screen of a trustor 1 (referred to as A1) in round 5. Trustor 1 is asked to choose RIGHT (withhold trust) or DOWN and select a participant B1, B2, B3, B4 (place trust in a specific trustee). The left-hand side of the screen shows a "history table" with 4 columns representing the 4 trustees (B1, B2, B3, and B4) and rows representing rounds. The current round, round 5, is indicated by the arrow "-->". In parentheses it is displayed which trustor (A) is at play in which round and for potential future rounds it is

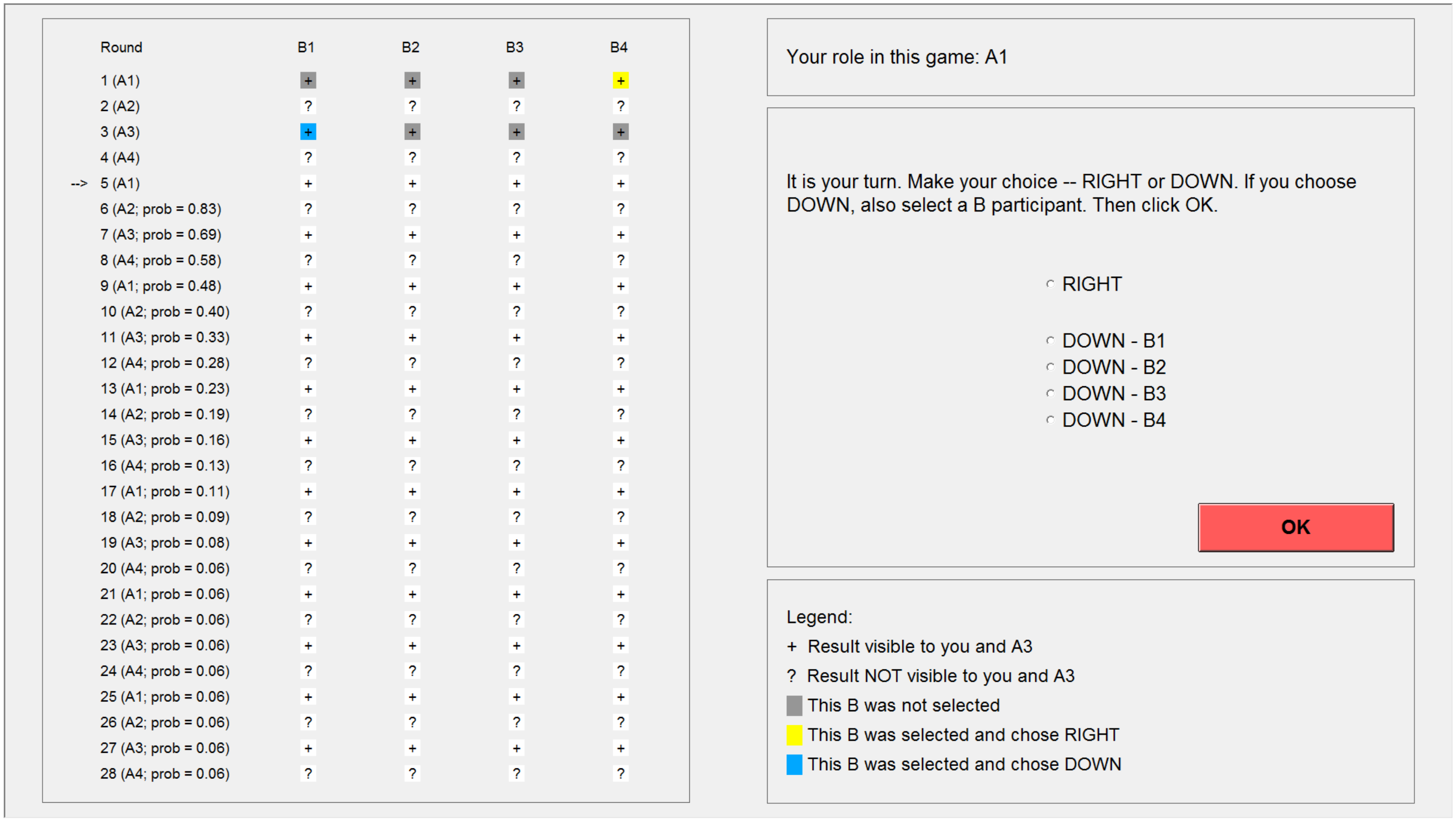

Supplementary Figure 1: Example screen for a trustor in the Partial condition.

also displayed what the probability is that the game reaches that round.*

In the Partial condition (as well as the Private condition), a trustor was shown + signs in the rounds for which he/she received or would receive information on trustor and trustee choices and question marks in the other rounds. Information on past choices was displayed by coloring the cells. Dark gray showed that a trustee was not chosen, yellow that a trustee was chosen and abused trust, and blue that a trustee was chosen and honored trust. In Supplementary Fig. 1, trustor 1 sees that he/she placed trust in trustee 4 (B4) in round 1 and that trustee 4 abused trust. Trustor 1 also sees that, in round 3, trustor 3 placed trust in trustee 1 who then honored trust. Trustor 1 does not see what happened in rounds 2 and 4. We note that in the Private condition, trustor 1 would in round 5 only see the outcome of round 1. In the Full condition, trustor 1 would see the outcomes of all past rounds.

Trustees were shown a similar screen, except that trustees always saw what happened in all past rounds. In the Private and Partial conditions, trustees also saw + signs and question marks, indicating about which past rounds the trustor currently at play had information and in which potential future rounds trustors would have information about the current round. Further information on the computer interface is found in the instructions that were distributed to participants and that are included below.

1.2.1 Instructions

We reprint an example of the instructions used in the experiment. Reprinted are the instructions from a session in which the first four games were played in the Full x Trust Problem condition and the last four games in the Private x Trust Problem condition. The instructions consist of two parts, one for each condition, that were handed out separately, prior to each condition. The reprinted figures were distributed on separate sheets. Instructions for other sessions are available from the authors.

*Because of a programming error the probabilities for reaching the last eight displayed rounds were shown incorrectly and were always the same as the probability of reaching round 20. In a quiz that subjects took after reading the instructions they had to look at an example screen of a game in round 5 and enter the probabilities for reaching rounds 7 and 9 (in two consecutive questions). The majority of subjects answered these questions correctly and no subject reported the discrepancy between the theoretical and displayed probabilities in the late rounds of the game when answering these questions or at the end of the session, suggesting that the error was probably not noticed and did not impact behavior. Also, because this error was made in all conditions, it did not confound our results.

Welcome and thank you for coming here!

The purpose of this experiment is to study decision making. Please do not communicate with other participants. Turn off your phone and put it away. Thank you very much. If at any point you have questions, raise your hand and we will assist you.

In this experiment, you will earn "points" by making decisions in "games." How much you earn depends on your decisions, the decisions of others and on chance. At the end of the session, you will be paid **1.5 US Dollar cents for every point** you earned.

All of the payments, participants, and other information that you read about in this study are real. Although some studies in other laboratories use fictional information, research in this laboratory focuses on studies of real situations. The following instructions tell you everything you need to know to earn as many points as possible and they are precisely the same for everyone in the room. Finally, everything is anonymous. No other participant will be able to link your decisions to your identity or get to know your name or earnings.

Description of the Game

The game is played in groups of 8 participants, four participants in the role of A (A1, A2, A3 and A4) and four participants in the role of B (B1, B2, B3 and B4). Before the game starts, you and the other participants are randomly separated into groups of 8 and each participant is randomly assigned his/her role and number. Throughout the game, all participants keep their role and number.

The Basic Interaction

The game proceeds in rounds. In each round only one of the As is active and interacts with the Bs. In round 1, it is A1's turn to interact with the Bs; in round 2, it is A2's turn, and so on such that in round 5, it is again A1's turn.

The As who are not active in a round get 30 points in that round. How many points the active A and the Bs get depends on their choices in the interaction shown in Figure 1 (see extra sheet). Please examine Figure 1 now. The active A (simply called "A" in Figure 1) chooses either "RIGHT" or "DOWN." If A chooses RIGHT, A gets 30 points and all four Bs get 30 points as well. If A chooses DOWN, A must select one of the four Bs (A must "SELECT B"). If A chooses DOWN and selects one of the Bs, the selected B participant chooses "RIGHT" or "DOWN." If the selected B chooses DOWN, A and the selected B get 50 points each. If the selected B chooses RIGHT, A gets nothing (0 points) and the selected B gets 80 points. In either case, the other Bs – the ones that were not selected by the A at play – get 30 points each, just as the As who are not active in this round.

The Duration of the Game

How many rounds the game lasts is determined randomly. It is as if we would roll a regular 6-sided die after every round and end the game if the outcome is a "6" but continue for at least one round if the outcome is not a "6." We let the computer do the "rolling of the die." Computer algorithms are never truly random but depend on the starting value used. We used the phone number of one of the researchers as the starting value. Anyway, all you need to know is that, for instance, if the game is in round 1, the probability that there will be a second round is $5/6 = 0.83$ and if the game is in round 7, the probability that there will be another round is also $5/6 = 0.83$.

The Computer Interface

All four As and Bs get informed about all choices. Have a look at Figures 2 and 3 (see extra sheets) that show the computer interface. What you will see on the right-hand side will be self-explanatory. On the left-hand side you see a "history window." Each of the four columns of plus signs ("+") represents one B participant and each row represents a round. The current round – round 5 on the example screens – is indicated by the arrow "-->". In parentheses it is displayed which A participant is at play in which round. For potential future rounds it is furthermore displayed what the probability is that the game does not end before this round. For example, given that the game is in round 5, the chance that the game does not end before round 7 is $0.83 * 0.83 = 0.69$. Hence, it is written "prob = 0.69" in round 7.

The color of the background of the plus signs of past rounds shows what choices were made. Dark-gray means that a B was not selected. Yellow means that B was selected and chose RIGHT. Blue means that B was selected and chose DOWN. If in a round the A-participant chooses RIGHT, the signs of all Bs are shown on a dark-gray background, as in round 4.

Should a game last more than 20 rounds, the history of the first rounds will disappear but you will still see the history of the 19 most recent rounds. Thus, you will always see at least 8 possible future rounds.

Organization of the Session

You will participate in 4 games, each lasting expectedly several rounds, one game played after the other. Then a small change to the rules of the game will be announced and then you participate again in 4 games. For each game you get randomly assigned to a new group of 8 participants and to your role. It is possible that you are with the same other participant in more than one game. However, should this happen, neither you nor the other participant will be able to notice this. Information about decisions

in earlier games will not be available to participants in later games.

Consent Forms and Quiz

If you wish to participate in this study, please read and sign the accompanying consent form; it explains your rights as a subject and the rules of confidentiality we adhere to. After signing the consent form, turn to the computer and answer a few questions that help you evaluate your understanding of the game.

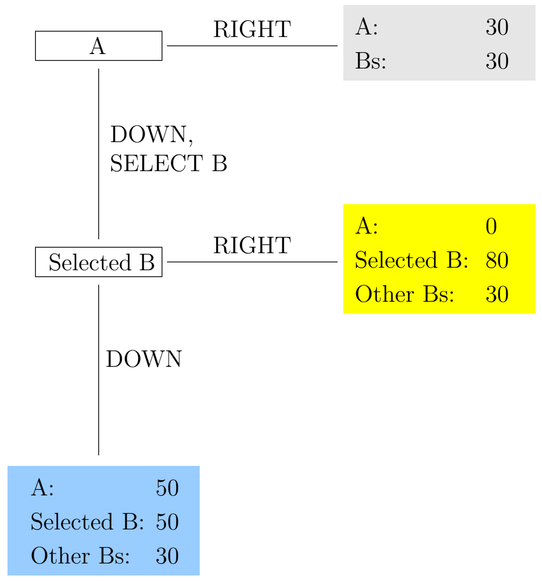

Figure 1: One round of play. The As who are not at play get 30 points each.

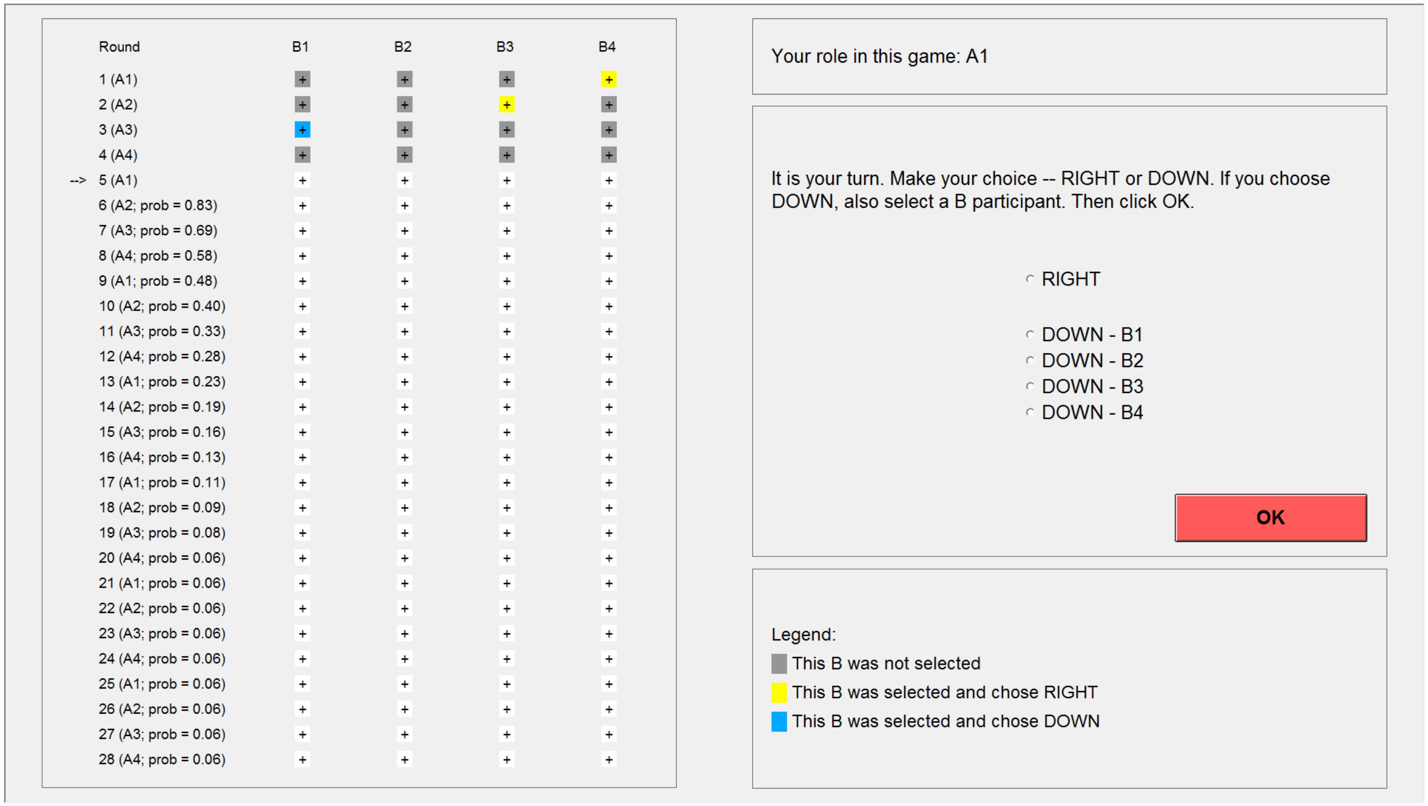

Figure 2: An Example Screen for an A

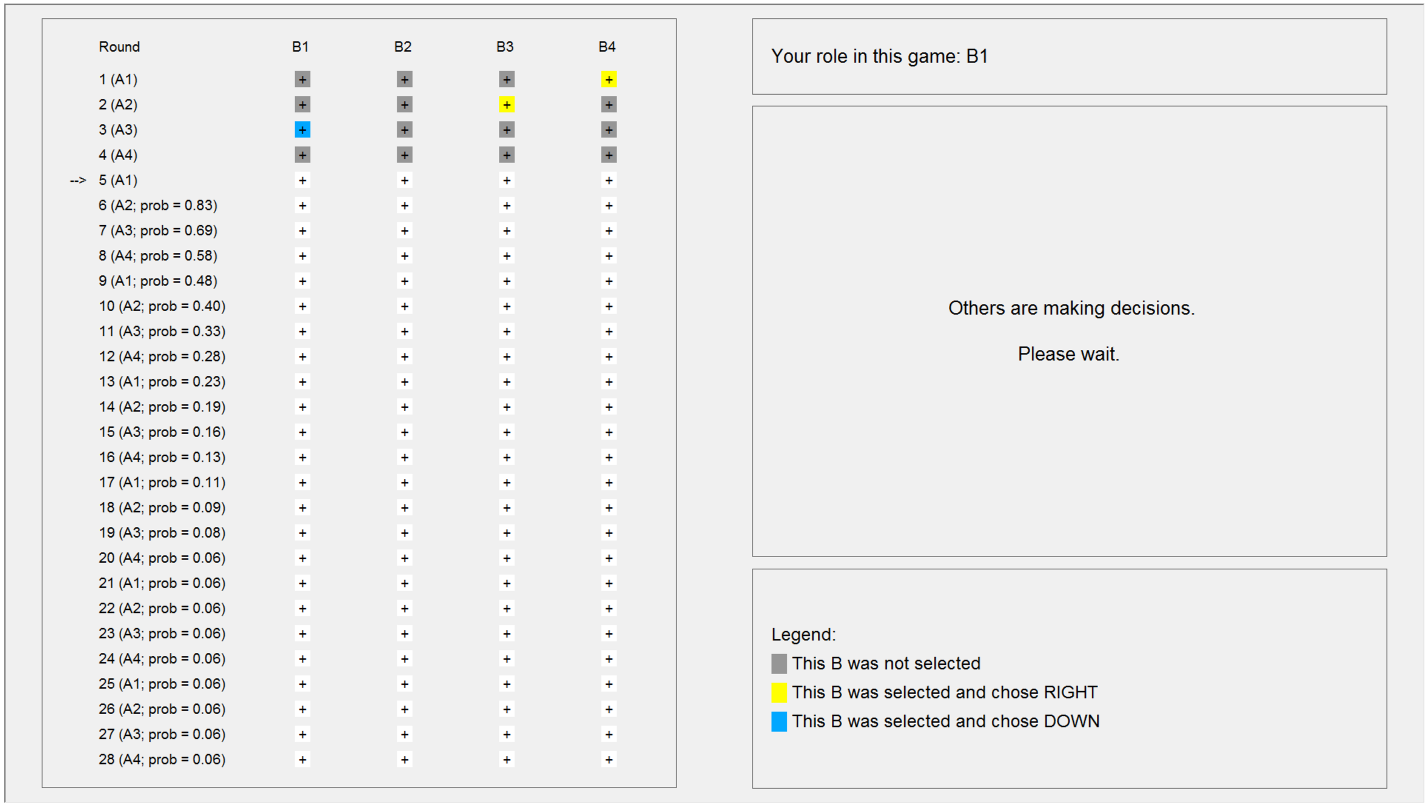

Figure 3: An Example Screen for a B

Instructions for Part 2

In the next four games, each A gets informed about the results of his/her own interactions but not about the results of the interactions of any other A. Take a moment to examine Figure 4, which shows an example screen for A1. A1 sees question marks ("?") in the rounds in which one of the other As is at play. The question marks of past rounds are not highlighted in color because A1 never receives information about the choices in these rounds.

The Bs still receive information about the outcome after every round and can see the entire history from the highlighting. To see this, examine Figure 5, which shows an example screen for B1. A participant in the role of a B sees plus signs ("+") and question marks in the same rows as the A who is at play (A1 in the example). The plus signs in past rounds indicate to a B which past rounds A has information about. The plus signs in potential future rounds show when it will be A's turn again. Note, the plus signs and question marks are placed in different rounds if it is a different A's turn.

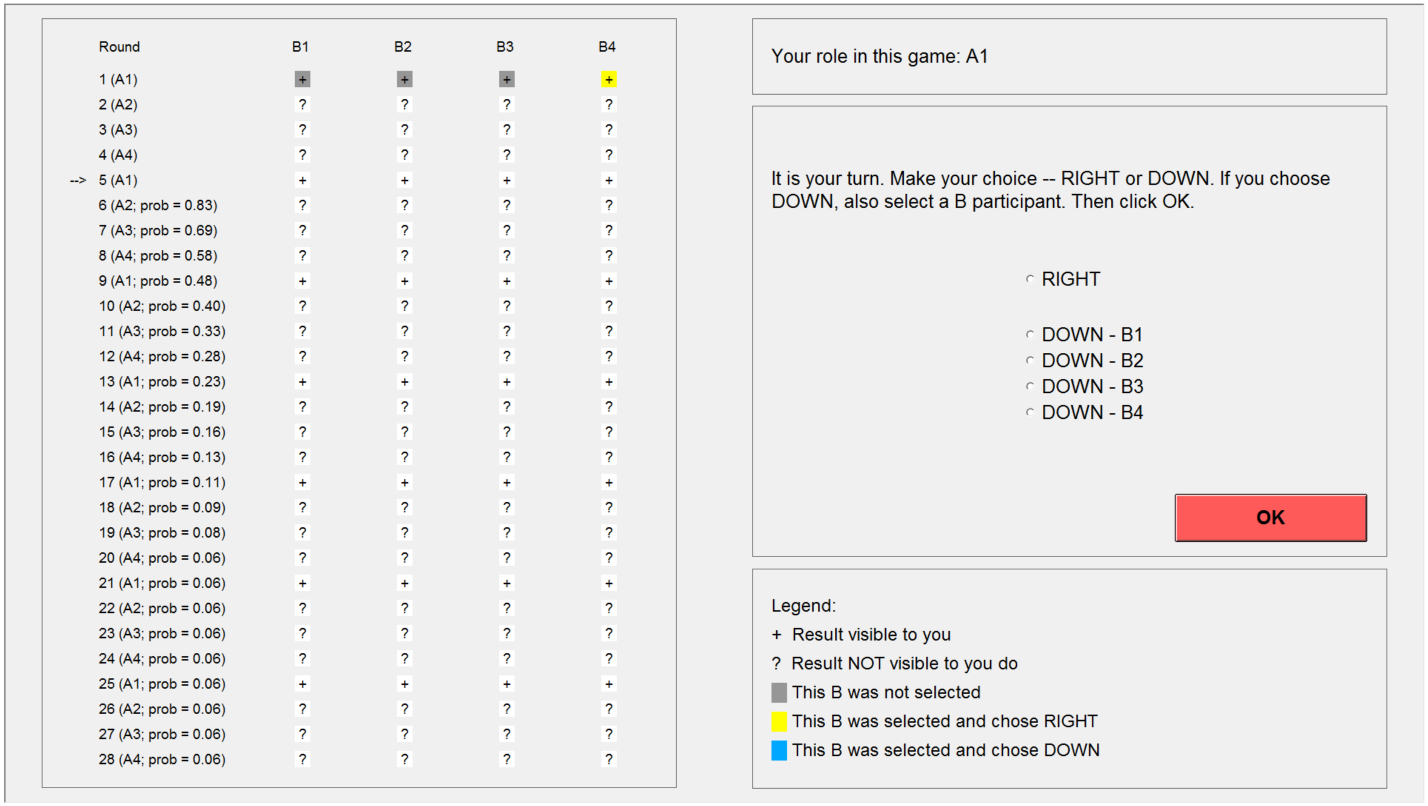

Figure 4: An Example Screen for an A

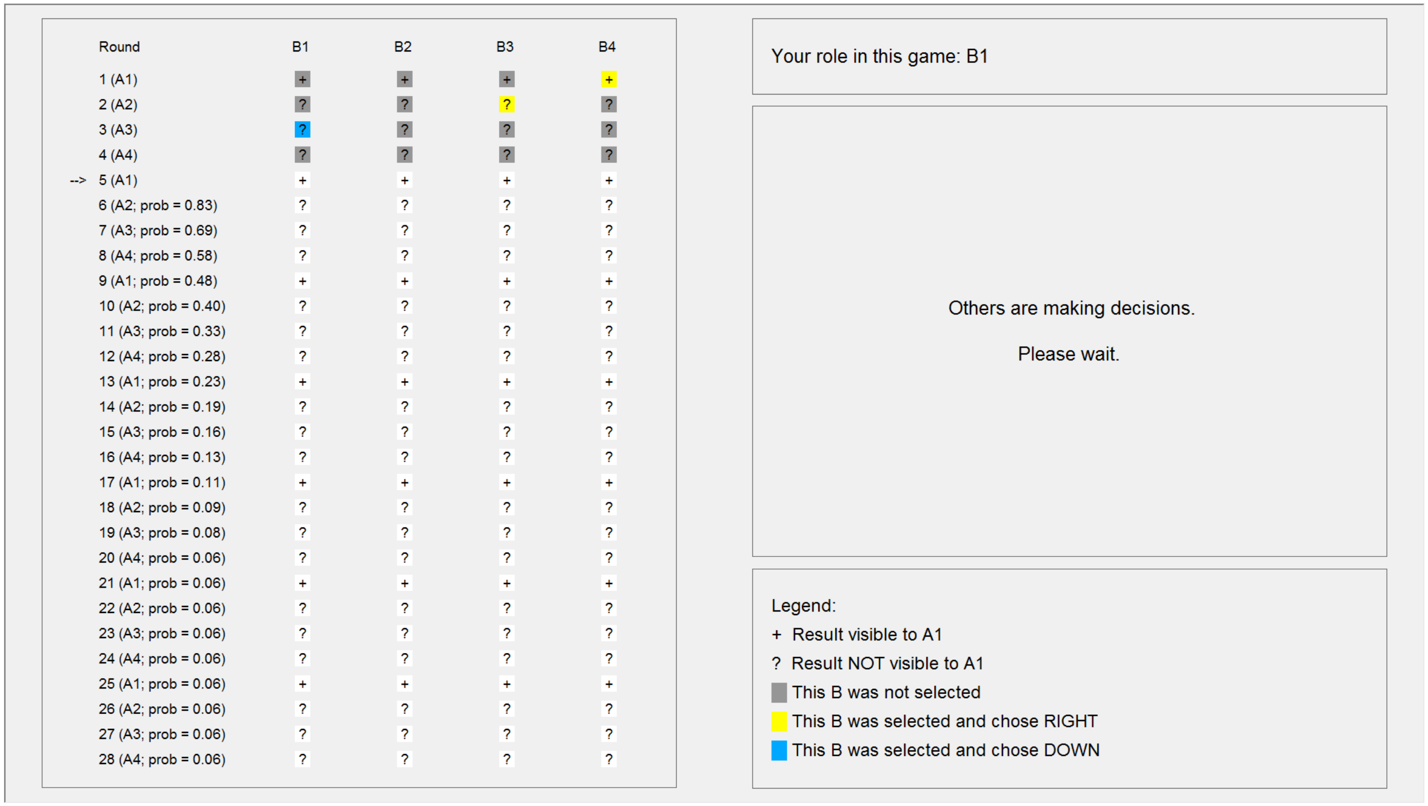

	Round	B1	B2	B3	B4
	1 (A1)	+	+	+	+
	2 (A2)	?	?	?	?
	3 (A3)	?	?	?	?
	4 (A4)	?	?	?	?
-->	5 (A1)	+	+	+	+
	6 (A2; prob = 0.83)	?	?	?	?
	7 (A3; prob = 0.69)	?	?	?	?
	8 (A4; prob = 0.58)	?	?	?	?
	9 (A1; prob = 0.48)	+	+	+	+
	10 (A2; prob = 0.40)	?	?	?	?
	11 (A3; prob = 0.33)	?	?	?	?
	12 (A4; prob = 0.28)	?	?	?	?
	13 (A1; prob = 0.23)	+	+	+	+
	14 (A2; prob = 0.19)	?	?	?	?
	15 (A3; prob = 0.16)	?	?	?	?
	16 (A4; prob = 0.13)	?	?	?	?
	17 (A1; prob = 0.11)	+	+	+	+
	18 (A2; prob = 0.09)	?	?	?	?
	19 (A3; prob = 0.08)	?	?	?	?
	20 (A4; prob = 0.06)	?	?	?	?
	21 (A1; prob = 0.06)	+	+	+	+
	22 (A2; prob = 0.06)	?	?	?	?
	23 (A3; prob = 0.06)	?	?	?	?
	24 (A4; prob = 0.06)	?	?	?	?
	25 (A1; prob = 0.06)	+	+	+	+
	26 (A2; prob = 0.06)	?	?	?	?
	27 (A3; prob = 0.06)	?	?	?	?
	28 (A4; prob = 0.06)	?	?	?	?

Figure 5: An Example Screen for a B

2 Data and analyses

2.1 Visual representation

Data from all 336 games are visualized in Supplementary Fig. 2. Each game is represented as a small light gray grid consisting of some number of rounds (rows) and four trustees (columns), similar as in the computer interface used in the experiment. Blue circles denote honored trust and orange triangles abused trust. The display of the games is organized by experimental conditions, with blocks of rows as reputation conditions and column blocks as trust conditions.†

Supplementary Fig. 2 shows that cascade-like patterns—series of honored trust repeatedly placed by all four trustors in a single trustee—frequently obtained in the Full x Trust Problem condition, as demonstrated by vertical strings of blue circles. In the Partial x Trust Problem condition, pairs of trustors often frequented two distinct trustees at the expense of the two other trustees, recognizable in Supplementary Fig. 2 as alternating diagonal patterns within a game. Such duopolistic motifs do not occur in the Full x Trust Problem condition. In both the Partial x Trust Problem and Full x Trust Problem condition, the cascades often formed before other trustees were given any opportunity to honor trust. Cascade-like patterns do not appear in the Private x Trust Problem condition, where trustors could not see the experiences of others, nor in the No Trust Problem conditions, in which abuse was costly for trustees. These raw data patterns form the basis of the results presented in the main text. Namely, trustors exhibit a strong tendency to imitate choices of others out of a desire to minimize the risk of trust abuse and not as a result of a general herding tendency (Fig. 1 main text), information sharing in trust situations leads to the emergence of inequality among trustees (Fig. 2 main text), and the selection of trustees is to some extent random, with potentially trustworthy trustees being excluded from exchange in an arbitrary manner (Fig. 3 main text).

2.2 Estimates‡

The prevalence of cascading (Fig. 1 of the paper): Figure 1 in the paper illustrates the prevalence of cascading as the proportion of times a trustor placing trust selected the trustee that had been selected on the last turn observed by the trustor, provided that

†In Supplementary Fig. 2, the games are furthermore arranged as blocks of three times four games separated by somewhat wider margins. Each of these blocks of three times four games shows data from the first or second sequence of four games played by three groups in a session.

‡We combine the data from the Trust Problem conditions with $T = 80$ and $T = 100$ for purposes of statistical power, see Section 3.3.

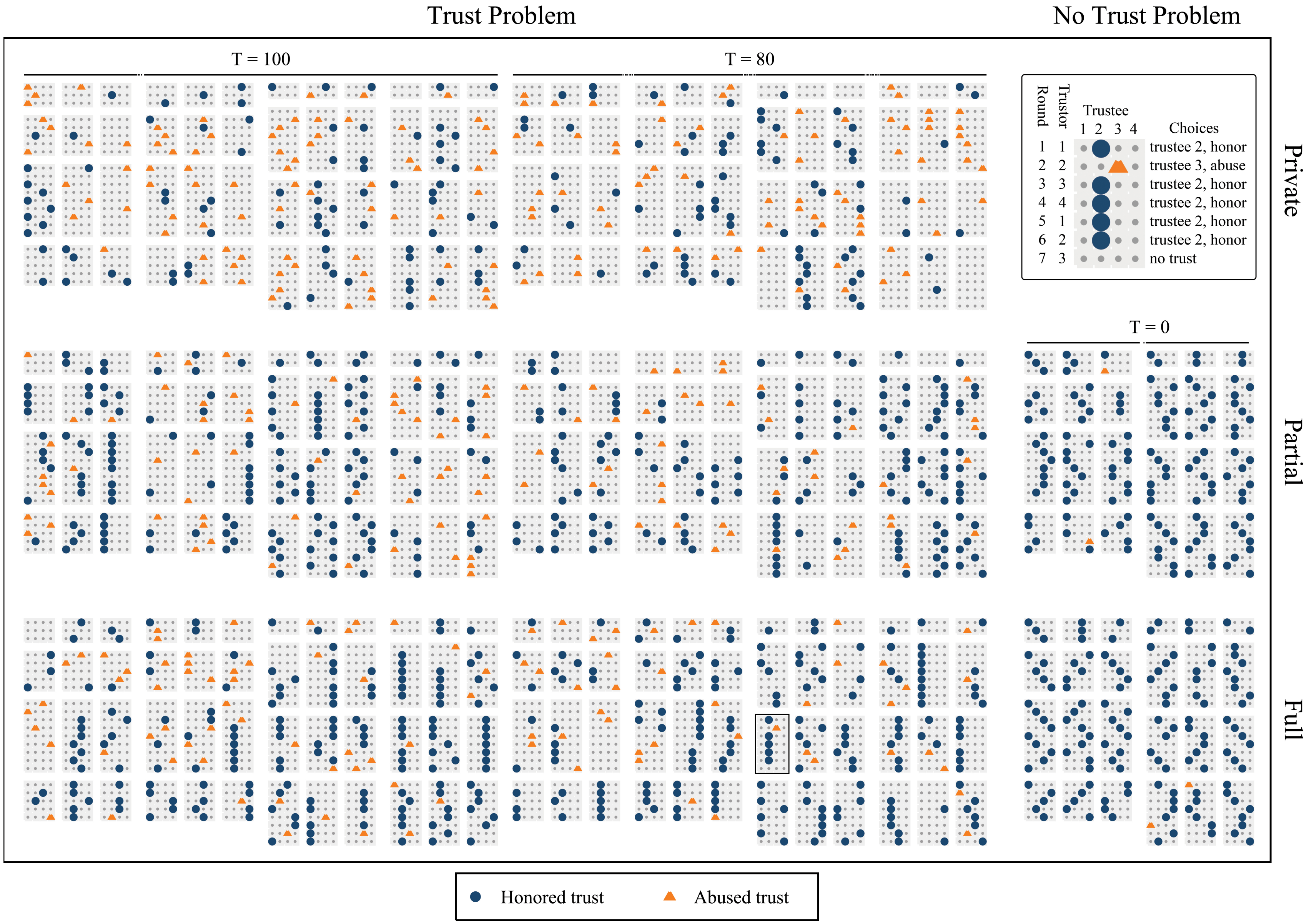

Supplementary Figure 2: Overview of the data: Play histories of the observed 336 games.

Supplementary Table 1: Cascade continuation by cascade length. Sample: Trustor choices of trustees in the Trust Problem condition ($T = 80, 100$) with Partial or Full information sharing.

Cascade length	# of Obs.	Percentage cascade continuation
1	416	59.4
2	142	66.2
3	50	74.0
4	18	88.9
5	7	100.0
6	3	100.0
7	1	100.0

trust had been honored. Fig. 1 is based on data from the Partial and Full conditions only, as the Private condition was not crossed with the No Trust Problem condition. Confidence intervals reported in Fig. 1 are obtained from logistic regression models with the dependent variable taking on value 1 if the trustor chooses the same trustee as was chosen on the last turn that the trustor observed, and 0 if the trustor chooses a different trustee. Observations are excluded if no previous turn was observed by the trustor or if trust was not placed or was abused on the last turn the trustor observed. The model contains only a constant α. The analyzed 608 choices are nested in 250 trustors and we used the clustered sandwich estimator of standard errors to adjust for possible correlations of error terms within individual trustors.[§] For the Trust Problem condition (416 choices by 202 trustors) $\alpha = .380$, 95% confidence interval: 0.151 to 0.608. For the No Trust Problem condition (192 choices by 48 trustors) $\alpha = -1.572$, 95% confidence interval: -2.111 to -1.034. Transforming these log-odds to probabilities shows, as illustrated in Fig. 1 in the main text, that the prevalence of repeated trustee choice (60%) was significantly higher than chance (25%) in the Trust Problem condition while in the No Trust Problem condition, the prevalence of repeated trustee choice (17%) was lower than expected by chance but not significantly so at the 95% level.

Supplementary Table 1 shows how the frequency of cascade continuation increased with cascade length, the number of consecutive turns a trustor could observe a trustee having been chosen and honoring trust. As Supplementary Table 1 shows, as the number of times trust was given to and honored by a given trustee increased, the probability of cascade continuation tended to increase as well.

[§]We used the implementation of the clustered sandwich estimator of standard errors in STATA (version 13, StataCorp LP, College Station, TX). See, e.g., ref. (47) on this estimator.

Supplementary Table 2: Linear regressions of the rate of honored trust and inequality among trustees on dummy variables for reputation conditions. Sample: Games played in the Trust Problem condition ($T = 80, 100$). Standard errors adjusted for clustering of games in 12 sessions (see footnote §).

	Inequality		Honored trust	
Partial	0.38	(0.35)	0.23**	(0.07)
Full	0.75*	(0.30)	0.28***	(0.06)
Intercept [Private]	0.52**	(0.16)	0.30***	(0.03)
Number of games	249		288	

Standard errors in parentheses
* $p < 0.05$, ** $p < 0.01$, *** $p < 0.001$

Effects of information sharing (Fig. 2 of the paper): Figure 2 in the paper shows how the reputation conditions affected inequality in the Trust Problem condition. Supplementary Table 2 reports the regression results illustrated in Fig. 2 as well as results from a regression model that establishes that information sharing led to higher levels of honored trust in the Trust Problem condition.

We measured inequality among trustees with the Modified Coefficient Of Variation[21] (MCOV). We obtained a maximum-likelihood estimate of the MCOV using a negative binomial regression of the counts of trust placed in each trustee. This measure of inequality is estimated only for the 249 games in the Trust Problem condition in which trust was placed more than once. The results of a regression of the MCOV on dummies for the conditions Partial and Full are reported in Supplementary Table 2 and show that inequality was significantly higher in the condition Full x Trust Problem than in the condition Private x Trust Problem.[¶] The estimates of inequality in the Partial and Full condition reported in Figure 2 of the paper are obtained by adding the respective coefficient to the intercept.

The second regression reported in Supplementary Table 2 shows that the rate of honored trust – the fraction of rounds of a game in which trust was placed and honored – was significantly higher at the 99% level in Partial x Trust Problem and Full x Trust Problem than in Private x Trust Problem.

Arbitrariness in trustee selection: We compared the disagreement in trustee choices in pairs of information-sharing trustors and non-information-sharing trustors in the Partial x Trust Problem condition. If trustee selection across pairs of information-sharing

[¶]Other common measures of inequality like the Gini Index or the Herfindahl Index also show greater inequality under more information sharing but their estimation is more problematic for small samples of count data.

trustors is arbitrary due to pair-specific histories of play, then disagreement should be higher among pairs of non-information-sharing trustors than among pairs of information-sharing trustors. To test whether this is the case, we measured the absolute difference in the number of times two trustors trusted a given trustee, summed across the four trustees. We then calculated for each game the difference in the average disagreement among pairs of non-information-sharing trustors (trustors 1 & 2, 1& 4, 2 & 3, and 3 & 4) and across pairs of information-sharing trustors (trustors 1 & 3 and 2 & 4). Call this difference D. The games that lasted only two rounds provide no information on disagreement among information-sharing trustors and we therefore calculated D only for 84 of the total of 96 games played in the Partial x Trust Problem condition. A linear regression of D on a constant shows that the difference D is significantly positive (constant $\alpha = 0.494$, $p = 0.007$; p-value adjusted for the nesting of observed games within sessions as explained in footnote §). That is, disagreement is indeed higher across non-information-sharing trustor pairs than across information-sharing trustor pairs, which indicates significant arbitrariness in the determination of which trustee becomes popular.

3 Theoretical Model

In this section, we demonstrate the production of arbitrary inequality in reputation systems game-theoretically in an indefinitely repeated trust game in which trustors can choose trustees. We describe the game in Subsection 3.1 and show in Subsection 3.2 that cascades emerge in game-theoretic equilibria in which trustors play a trigger strategy[48]. The Trust Problem conditions of our experiment implemented a specific case of the game. Subsection 3.3 contains remarks on how we determined the parameters for the experiment from the theoretical model.

3.1 The game

In our game, $N_1 > 1$ trustors and $N_2 > 1$ trustees interact in consecutive rounds 1, 2, 3, ... The trustors take turns; trustor i plays in rounds i, $i + N_1$, $i + 2N_1$, etc. After every round, the next round gets played with probability $0 < w < 1$ while the game ends with probability $1 - w$.

Every round, the trustor i in turn chooses whether to withhold trust or to select one of the trustees and place trust in that trustee. If trustor i does not place trust in any trustee, i receives payoff P_1, the same payoff that any trustor not in turn receives. Any trustee who is not selected by trustor i receives payoff P_2. If trustor i chooses to place

trust in trustee j, then j can choose whether to honor or abuse trust. If j honors trust, the payoffs for i and j are $R_1 > P_1$ and $R_2 > P_2$, respectively. If j abuses trust, i's and j's payoffs are $S_1 < P_1$ and T_j, respectively.

Trustees differ in how much they earn when abusing trust, i.e., in T_j, but throughout the repeated game, T_j stays the same. T_j is drawn independently for each trustee j before round 1 from a probability distribution with unbounded density $\mathbf{F}$. While $\mathbf{F}$ is common knowledge, the actual manifestation of T_j is private information of trustee j.

Reputation systems are operationalized as subsets of trustors sharing information on outcomes of prior rounds. The N_1 trustors are divided into n equally sized, disjoint information sharing communities, where $1 \leq n \leq N_1$. While trustees are always informed about all past choices, trustors have only information about the choices made in past rounds in which a trustor of their own information sharing community was at play.

The trustors of different information sharing communities alternate in taking decisions. That is, some trustor from community c plays in rounds c, $n+c$, $2n+c$, $\ldots$. In sum, each trustor plays in every N_1^{th} round and every n^{th} round is played by a trustor from some given information sharing community.

3.2 Analysis

We identify conditions for equilibria involving honored trust. We focus analysis on a strategy for trustors that is associated with the least restrictive condition for such equilibria (see below). We call this strategy "*search & trigger strategy*" and first introduce some notation to define it. We let H_{it} denote the set of trustworthy or **h**onorable trustees—trustees about whom trustor i at the beginning of round t has information on at least one instance of honoring trust and no instance of abusing trust. We let B_{it} denote the set of unknown or **b**lank trustees—trustees about whom i has no information on past behavior at the beginning of round t.

Definition 1. *The search & trigger strategy of a trustor.*

- *If $H_{it} = \varnothing$ and $B_{it} \neq \varnothing$, trustor i places trust with probability $\gamma > 0$ in a trustee that she chooses uniformly at random from B_{it} and withholds trust with probability $1 - \gamma$.*

- *If $H_{it} = \varnothing$ and $B_{it} = \varnothing$, trustor i withholds trust.*

- *If $H_{it} \neq \varnothing$, trustor i places trust in a trustee that she chooses uniformly at random from H_{it}.*

We restrict the analysis to symmetric equilibria in the sense that we require that γ is the same for all trustors. Allowing for asymmetric equilibria would not change any of the results relevant to the claims in our paper.

A trustee's best response to the search & trigger strategy of trustors is to either always honor trust or to always abuse trust when trusted. Lemma 1 specifies the threshold for trustworthiness and the proportion of trustees that will be trustworthy.

Lemma 1. *A trustee j's best response to the search & trigger strategy of trustors is to honor trust when selected if and only if*

$$w^n \geq \frac{T_j - R_2}{T_j - P_2} \Leftrightarrow T_j \leq \frac{R_2 - w^n P_2}{1 - w^n}. \tag{1}$$

So the proportion ρ of trustees who honor trust when selected is

$$\rho = \int_0^{\frac{R_2 - w^n P_2}{1 - w^n}} d(T_j). \tag{2}$$

Proof. Lemma 1 is a straightforward extension of a well-known result from the analysis of indefinitely repeated trust games played between one trustor and one trustee. It can be shown that if, in such a game, the trustor plays a trigger strategy (places trust in every round as long as the trustee always honored trust, and never places trust again after a first abuse) the trustee's best response is to withstand the temptation of trust abuse and always honor trust if the continuation probability w is at least as large as $(T_j - R_2)/(T_j - P_2)$, i.e., if $w \geq (T_j - R_2)/(T_j - P_2)$, see e.g., ref. 48). If, in our model, the trustors play the search & trigger strategy, current trustworthiness leads to future trust and abuse to no trust. However, a trustee's behavior vis-à-vis one trustor gets sanctioned exclusively in those future rounds in which that trustor or another trustor of the same information sharing community is at play. Specifically, with a trustor from a given information sharing community being at play in every n^{th} round, a trustee will be punished or rewarded for his current behavior only in every n^{th} future round. To withstand the short-term incentive for trust abuse, a trustee needs to be more strongly incentivized when n is greater and it is a best-response for a trustee j to always honor trust only if $w^n \geq \frac{T_j - R_2}{T_j - P_2}$. □

Proposition 1 specifies the condition under which there exists an equilibrium in which the trustors play the search & trigger strategy. If the condition that Proposition 1 specifies holds, the search & trigger strategy induces a large enough proportion ρ of trustees to be trustworthy such that it is a best response for each trustor i to "search" (place trust in a blank trustee), if $H_{it} = \varnothing$, $B_{it} \neq \varnothing$, and provided that other trustors use the same

probability $\gamma > 0$. If the condition in Proposition 1 does not hold, rational trustors will never place trust because the potential benefit of finding an honorable trustee does not warrant the risk of trust abuse when placing trust in a blank trustee.

Proposition 1. *There exists a Nash equilibrium in which all trustors play the search & trigger strategy with the same probability γ and some portion ρ of trustees honor trust when selected if and only if*

$$\frac{P_1 - S_1}{R_1 - S_1} \leq \frac{\rho}{1 - w^{N_1}(1-\rho)}, \tag{3}$$

where ρ is the proportion of trustees who honor trust when selected as specified in Lemma 1.

Proof. To prove Proposition 1, we need to establish that playing the search & trigger strategy with some positive search probability γ is a best-response for a trustor i if Eq. (3) in Proposition 1 holds (and given that the other trustors play the search & trigger strategy with the same search probability γ and trustees play their best-response to this strategy as specified in Lemma 1). Three claims need to be established: (1) that it is a best-response for trustor i to search with some positive probability γ if Eq. (3) holds and i does not know an honorable trustee but knows blank trustees ($H_{it} = \varnothing$ and $B_{it} \neq \varnothing$), (2) that i should place trust in an honorable trustee if i knows such a trustee ($H_{it} \neq \varnothing$), and (3) that i should withhold trust if i has information on some past abuse of all N_2 trustees ($H_{it} = \varnothing$ and $B_{it} = \varnothing$).

We omit a formal proof of claims (2) and (3). That a trustee either always honors trust or always abuses trust, depending on T_j, (see Lemma 1) implies straightforwardly that claims (2) and (3) hold irrespectively of the specific parameters of the game. It furthermore implies for claim (1) that it can never pay off for a trustor i to deviate from searching (placing trust in a blank trustee) by instead placing trust in a trustee of which i knows of a past abuse.

For claim (1) to hold, it must additionally not pay off for trustor i to deviate from searching with probability γ by strictly withholding trust. This could pay off as it allows avoiding the risk of trust abuse. A rational trustor will only search if the expected short-term costs of searching does not exceed the expected long-term benefit of searching. To establish the condition under which this is the case, we apply the one-shot deviation principle[49]: We calculate trustor i's costs and benefits of searching in round t under the assumption that i plays the search & trigger strategy with the same probability γ as all other trustors in all rounds following round t. We calculate these costs and benefits without fixing a specific γ, assuming only that γ is larger than 0 and the same for all trustors.

Consider first the costs of searching. In round t, i's expected payoff from searching (placing trust in a blank trustee) with probability γ is $\gamma(\rho R_1 + (1-\rho)S_1) + (1-\gamma)P_1$ while i's payoff from strictly withholding trust is P_1. If $(P_1 - S_1)/(R_1 - S_1) < \rho$, there are no search costs: i's expected payoff for round t is larger if i searches than if i strictly withholds trust. If $(P_1 - S_1)/(R_1 - S_1) > \rho$, on the other hand, i has an immediate expected search cost in round t from searching with probability γ of size

$$\gamma(P_1 - S_1) - \gamma\rho(R_1 - S_1). \tag{4}$$

Searching can still be beneficial as it increases the chance of knowing an honorable trustee in the future. If i strictly withholds trust in round t and then reverts to playing the search & trigger strategy, the probability that i knows an honorable trustee in round $t + fN_1$, the fth future round in which i would be at play, is $1 - (1-\gamma\rho)^{f\frac{N_1}{n}-1}$. In the calculation of this probability, $(1-\gamma\rho)$ is the probability that an honorable trustee is *not* found in a round in which the trustor at play searches with probability γ; $(1-\gamma\rho)^{f\frac{N_1}{n}-1}$ is the probability that no honorable trustee is found over the $f\frac{N_1}{n} - 1$ rounds in which one of the $\frac{N_1}{n}$ trustors of i's information sharing community is at play between round t and round $t + fN_1$. On the other hand, if i does search with probability γ in round t, the probability that i knows an honorable trustee in round $t+fN_1$ is $1-(1-\gamma\rho)^{f\frac{N_1}{n}-1}(1-\gamma\rho) = 1 - (1-\gamma\rho)^{f\frac{N_1}{n}}$. Hence, if i searches in round t with probability γ, this increases the probability that i knows an honorable trustee in round $t + fN_1$ by

$$\Big(1 - (1-\gamma\rho)^{f\frac{N_1}{n}}\Big) - \Big(1 - (1-\gamma\rho)^{f\frac{N_1}{n}-1}\Big) = \gamma\rho(1-\gamma\rho)^{f\frac{N_1}{n}-1}. \tag{5}$$

In round $t + fN_1$, i receives R_1 if he knows an honorable trustee and $\gamma\big(\rho R_1 + (1-\rho)S_1\big) + (1-\gamma)P_1$ if he does not know an honorable trustee, given that i plays the search & trigger strategy in that round. Thus, i's benefit in round $t + fN_1$ of knowing an honorable trustee is

$$(1-\gamma\rho)(R_1 - S_1) - (1-\gamma)(P_1 - S_1). \tag{6}$$

The expected benefit that i has in round $t + fN_1$ from searching in round t is obtained from multiplying Eq. (5) with Eq. (6)—multiplying the increase in the probability of knowing an honorable trustee with the value of knowing an honorable trustee. The expected total long-term benefit that i derives from searching in round t is obtained by summing up the expected benefits over potential future rounds in which i would be at play, multiplied with the probability that these rounds are reached. This yields an expected

long-term benefit to i from searching in round t with probability γ of

$$\sum_{f=1}^{\infty} w^{fN_1}\gamma\rho(1-\gamma\rho)^{f\frac{N_1}{n}-1}\Big((1-\gamma\rho)(R_1-S_1)-(1-\gamma)(P_1-S_1)\Big). \tag{7}$$

It is then a best-response for trustor i to search with probability γ in round t if and only if the immediate cost of searching (Eq. (4)) does not exceed the expected long-term benefit from searching (Eq. (7)). That is, if

$$\begin{aligned}
&\gamma(P_1-S_1)-\gamma\rho(R_1-S_1)\leq \\
&\quad\sum_{f=1}^{\infty} w^{fN_1}\gamma\rho(1-\gamma\rho)^{f\frac{N_1}{n}-1}\Big((1-\gamma\rho)(R_1-S_1)-(1-\gamma)(P_1-S_1)\Big) \\
&\Leftrightarrow \frac{P_1-S_1}{R_1-S_1}\leq\frac{\rho}{1-w^{N_1}(1-\rho)(1-\gamma\rho)^{\frac{N_1}{n}-1}}.
\end{aligned} \tag{8}$$

If Eq. (8) holds, it is a best-response for each trustor i to search with the same probability $\gamma > 0$ as other trustors if $H_{it} = \varnothing$ and $B_{it} \neq \varnothing$. Thus, if Eq. (8) holds, claim (1) holds and there exists an equilibrium in which all trustors play the search & trigger strategy with the same probability γ. If Eq. (8) does not hold, no such equilibrium exists. A rational trustor i would deviate from searching with probability γ by strictly withholding trust because the expected gain of searching does not warrant the risk of trust abuse in the search.

Eq. (8) implies Eq. (3) in Proposition 1 as the condition for the existence of an equilibrium in which the trustors play the search & trigger strategy. For $n = N_1$ (all trustors are isolates), Eq. (8) reduces to Eq. (3). For $n < N_1$, Eq. (8) reduces to Eq. (3) as γ goes to 0. That is, if $n < N_1$ and Eq. (3) holds, γ can be chosen such that Eq. (8) holds.[‖] □

[‖]Searching a trustworthy trustee can resemble a Volunteer's Dilemma[50]. From the proof of Proposition 1 one can infer that it is possible that there exists an equilibrium in which $\gamma = 1$ if there is no information sharing ($n = N_1$) while only equilibria in which $0 < \gamma < 1$ exist if there is information sharing ($n < N_1$). That is, given γ, Eq. (8) is more restrictive if n is smaller (one could intervene that the proportion of trustworthy trustees, ρ, tends to increase in information sharing; however, as we did not assume a specific distribution $\mathbf{F}$, ρ may increase only marginally or even remain constant). That the condition for an equilibrium in which $\gamma = 1$ can become more restrictive with (more) information sharing reflects that the search efforts of information sharing partners of a trustor i diminish the effect of i's own search on the probability of knowing a trustworthy trustee in the future. If there is information sharing and an equilibrium in which $\gamma = 1$ would only exist if there was no information sharing, searching a trustworthy trustee resembles a Volunteer's Dilemma: A trustor prefers to search if none of her information sharing partners will search but if all her information sharing partners will search with probability 1, she prefers not to search. In that situation, an equilibrium exists such that each trustor i trustor searches with the same "small enough" probability γ, chosen such that each trustor is indifferent between searching and not

We conclude the theoretical analysis with explaining, first, how the number of information sharing communities, n, into which the trustors are fragmented affects whether there exists an equilibrium involving honored trust. Second, we discuss how arbitrary inequality emerges in an equilibrium in which the trustors play the search & trigger strategy and how the degree of arbitrary inequality depends on n.

Our model reproduces the well-known result that information sharing can make honored trust possible when honored trust would not be possible without information sharing. Note, first, that the search & trigger strategy leads to the least restrictive possible condition for an equilibrium involving honored trust. It implies the most severe punishment for trust abuse (no more trust from any trustor of the information sharing community of an abused trustor) and the largest reward for honoring trust (continued trust from all trustors of the information sharing community of a focal trustor). Sanctioning of trustees by trustors outside of a focal information sharing community is impossible. Therefore, if the search & trigger strategy does not render honoring trust a best response for trustee j, no other strategy of the trustors will. The search & trigger strategy thus maximizes the proportion of trustworthy trustees ρ and, hence, minimizes the likelihood of trust abuse when placing trust in a blank trustee. By playing the search & trigger strategy, trustors also take maximum advantage of knowing a trustworthy trustee and are thus maximally incentivized to search in the first place. Hence, if Eq. (3) does not hold, there cannot be an equilibrium involving honored trust.

The claim that information sharing facilitates trust then derives from the fact that Eq. (3) may hold for a small number n of information sharing communities but not for a larger n. The condition in Eq. (3) depends on n indirectly via ρ. The proportion of trustworthy trustees ρ increases weakly if n becomes smaller (compare Eq. (1) in Lemma 1). As ρ increases, Eq. (3) becomes less restrictive ($\partial(\rho/(1 - w^{N_1}(1 - \rho)))/\partial\rho = (1-w^{N_1})/(1-w^{N_1}(1 - \rho))^2 > 0$), which reflects that, as ρ increases, the likelihood of trust abuse when searching for a trustworthy trustee becomes smaller. Thus, there may be a critical value for the number of information sharing communities, n^*, such that an equilibrium involving honored trust exists if $n \leq n^*$ but not if the trustors are fragmented into more information sharing communities ($n > n^*$).

Now consider the emergence of inequality among trustees in how often they get trusted. Trustees making the same choice on every round together with trustors' search & trigger strategy gives rise to reputation cascades: Once the trust of a trustor from some information sharing community is honored by some trustee, all trustors of this community place trust in that trustee in all subsequent rounds. Hence, becoming the first trustee

searching, i.e., such that Eq. (8) holds with equality.

j with $T_j \leq \frac{R_2 - w^n P_2}{1-w^n}$ to be trusted by a trustor of some information sharing community yields that trustee all future trust of that informaion sharing community. The resulting inequality among "trustworthy trustees", trustees with $T_j \leq \frac{R_2 - w^n P_2}{1-w^n}$, should be considered "arbitrary", because trustor earnings do not depend on which of these trustees happen to be chosen. Arbitrary inequality is greater when the number of information sharing communities n is smaller because if n is smaller, fewer trustworthy trustees get trusted in more of the rounds while more trustworthy trustees are excluded.** Inequality in the placements of trust across all N_2 trustees (including untrustworthy trustees) also increases as n decreases. Untrustworthy trustees are at the lower end in terms of how often they get trusted and if n is smaller, fewer of them will ever get trusted and they are expected to be trusted less often because when information sharing groups are larger, fewer search trials are needed until all trustors know a trustworthy trustee.

3.3 Determination of experimental parameters

While our theoretical model assumes heterogeneity among trustees in the payoff for trust abuse (T_j), we did not induce such heterogeneity in our experimental design. The monetary payoff for trust abuse was the same for every trustee in a session ($T_j = T$). Instead we relied on intrinsic heterogeneity among subjects in trustworthiness, for example, due to variability in social preferences. This strategy allowed us to keep the experiment simple and readily understandable to subjects, and prevented variation in payoffs across trustees from becoming artificially salient in subjects' experiences.

We sought to create an experimental environment in which reputation systems promote trust. Not knowing the social preferences of subjects we implemented two distinct values of T in our attempt to accomplish this. We chose values of T such that conditions would vary in whether honoring trust by a trustee without social preferences constitutes a best response to trustors' search & trigger strategy. With $T = 80$, honoring trust is a best response in Partial and Full, but not in Private. With $T = 100$, honoring trust is a best response only in Full. As it turned out, both $T = 80$ and $T = 100$ ended up creating the sought-after environment by producing more honored trust in Partial and Full compared with Private. The two values did not yield significantly different levels of honored trust or inequality. In the presentation of the results we jointly analyze data from games with $T = 80$ and $T = 100$ for purposes of statistical power.

**In addition, when n is smaller, more trustees who would be trustworthy go empty-handed, because when n is smaller, the threshold for trustworthiness is smaller (see (Eq. 1)) and, hence, more trustees will be trustworthy.

References

21. Allison, P. D. Estimation and testing for a Markov model of reinforcement. *Sociol. Methods Res.* **8,** 434–453 (1980).

45. Huck, S., Lünser, G. K. & Tyran, J.-R. Competition fosters trust. *Game. Econ. Behav.* **76,** 195–209 (2012).

46. Fischbacher, U. z-tree: Zürich toolbox for ready-made economic experiments. *Exp. Econ.* **10,** 171–178 (2007).

47. Rogers, W. H. Regression standard errors in clustered samples. *Stata Tech. Bull.* **13,** 19–23 (1994).

48. Friedman, J. W. *Game Theory with Applications to Economics* (Oxford University Press, Oxford, 1986).

49. Mailath, V. & Samuelson, L. *Repeated Games and Reputations* 24–28 (Oxford University Press: Oxford, 2006).

50. Diekmann, A. Volunteer's dilemma. *J. Conflict Resolut.* **29,** 605–610 (1985).
